# Comparative Analysis of anti-Shine- Dalgarno Function in *Flavobacterium johnsoniae* and *Escherichia coli*

**DOI:** 10.3389/fmolb.2021.787388

**Published:** 2021-12-13

**Authors:** Zakkary A. McNutt, Mai D. Gandhi, Elan A. Shatoff, Bappaditya Roy, Aishwarya Devaraj, Ralf Bundschuh, Kurt Fredrick

**Affiliations:** ^1^ Ohio State Biochemistry Program, The Ohio State University, Columbus, OH, United States; ^2^ Center for RNA Biology, The Ohio State University, Columbus, OH, United States; ^3^ Department of Microbiology, The Ohio State University, Columbus, OH, United States; ^4^ Department of Physics, The Ohio State University, Columbus, OH, United States; ^5^ Department of Chemistry and Biochemistry, The Ohio State University, Columbus, OH, United, States; ^6^ Division of Hematology, Department of Internal Medicine, The Ohio State University, Columbus, OH, United States

**Keywords:** ribosome, translation, RF2 (prfB), initiation, bacteroidetes

## Abstract

The anti-Shine-Dalgarno (ASD) sequence of 16S rRNA is highly conserved across Bacteria, and yet usage of Shine-Dalgarno (SD) sequences in mRNA varies dramatically, depending on the lineage. Here, we compared the effects of ASD mutagenesis in *Escherichia coli*, a Gammaproteobacteria which commonly employs SD sequences, and *Flavobacterium johnsoniae*, a Bacteroidia which rarely does. In *E. coli*, 30S subunits carrying any single substitution at positions 1,535–1,539 confer dominant negative phenotypes, whereas subunits with mutations at positions 1,540–1,542 are sufficient to support cell growth. These data suggest that CCUCC (1,535–1,539) represents the functional core of the element in *E. coli*. In *F. johnsoniae*, deletion of three ribosomal RNA (*rrn*) operons slowed growth substantially, a phenotype largely rescued by a plasmid-borne copy of the *rrn* operon. Using this complementation system, we found that subunits with single mutations at positions 1,535–1,537 are as active as control subunits, in sharp contrast to the *E. coli* results. Moreover, subunits with quadruple substitution or complete replacement of the ASD retain substantial, albeit reduced, activity. Sedimentation analysis revealed that these mutant subunits are overrepresented in the subunit fractions and underrepresented in polysome fractions, suggesting some defect in 30S biogenesis and/or translation initiation. Nonetheless, our collective data indicate that the ASD plays a much smaller role in *F. johnsoniae* than in *E. coli*, consistent with SD usage in the two organisms.

## Introduction

Faithful protein synthesis requires that the translation machinery select the correct start codon over other AUG or similar trinucleotides. In all cells, intrinsic sequence and structural features of the mRNA enable start codon recognition. One well-known feature in prokaryotic cells is the Shine-Dalgarno (SD) sequence, a purine-rich element that lies upstream from the start codon and can pair with the anti-SD (ASD) sequence contained in the 3’ tail of 16S rRNA (Shine and Dalgarno, 1974; Steitz and Jakes, 1975). SD-ASD interaction helps position the start codon in the 30S subunit P site during initiation (Vellanoweth and Rabinowitz, 1992; Studer and Joseph, 2006; Hussain et al., 2016). In *Escherichia coli*, most mRNAs contain a SD (Nakagawa et al., 2017), and numerous genetic studies underscore the functional importance of the SD in such mRNAs (Hui and de Boer, 1987; Jacob et al., 1987; de Smit and van Duin, 1994). At the same time, there are many mRNAs that naturally lack a SD and yet are accurately and efficiently translated, indicating that other features of mRNA can direct start codon selection (Espah Borujeni et al., 2014; Li et al., 2014; Hockenberry et al., 2017).

Genomic studies have revealed that SD usage varies dramatically across Bacteria (Nakagawa et al., 2010; Accetto and Avguštin, 2011; Nakagawa et al., 2017). Certain lineages, such as Bacteroidia (formerly Bacteroidetes), generally lack SD sequences. Baez *et al.* analyzed translation in *Flavobacterium johnsoniae*, a member of Bacteroidia, to understand how start codon selection occurs in these organisms (Baez et al., 2019). They found that reduced secondary structure, a Kozak-like sequence (A-3, A-6), and an upstream A-motif (A-12, A-13) contribute to initiation in *F. johnsoniae*. Additionally, they showed that, across the Bacteroidia, AUG trinucleotides in the vicinity of the start codon are clearly underrepresented. Thus, elimination of alternative AUG trinucleotides in the translation initiation region (TIR) is one means by which these organisms compensate for the absence of SD-ASD pairing (Baez et al., 2019).

Variable usage of SD sequences in Bacteria came as a surprise, because the ASD is highly conserved across the entire domain (Cannone et al., 2002). Reporter gene studies in several representative organisms have shown that Bacteroidia ribosomes fail to recognize SD sequences in the cell (Accetto and Avguštin, 2011; Wegmann et al., 2013; Mimee et al., 2015), as though the ASD is functionally occluded in some way. A recent cryo-EM structure of the *F. johnsoniae* ribosome at 2.8 Å resolution uncovered the basis of ASD inhibition (Jha et al., 2021). The 3′ tail of 16S rRNA binds a pocket formed by bS21, bS18, and bS6 on the 30S platform domain, physically sequestering the ASD nucleotides. Residues of these proteins that interact with the 3’ tail are uniquely conserved in the Bacteroidia, suggesting that the mechanism of ASD occlusion is conserved across the class (Jha et al., 2021).

Interestingly, SD sequences are absent from most but not all Bacteroidia genes. In fact, ribosomal protein genes *rpsU* (bS21) and/or *rpsR* (bS18) often contain a “strong” SD, depending on the organism order (Jha et al., 2021). The corresponding proteins, bS21 and bS18, contribute to the mechanism of ASD occlusion, as mentioned above. This implies some type of translational autoregulation, the details of which remain to be elucidated. In Flavobacteriales, SDs are especially rare, and *rpsU* (bS21) is the only ribosomal gene to harbor one. A subset of Flavobacteriales, including Chryseobacteria and related species, has the alternative ASD sequence 5′-UCUCA-3′ rather than the canonical ASD (5′-CCUCC-3′). Remarkably, compensatory substitutions are seen upstream of *rpsU* in these organisms, indicative of natural covariation. Thus, translation of at least one gene, *rpsU*, entails SD-ASD pairing in the Flavobacteriales (Jha et al., 2021).

In this study, we compare the effects of ASD mutations in *E. coli* and *F. johnsoniae*. In *E. coli*, any single substitution of nucleotides 1,535–1,539 confers a dominant negative phenotype, defining the functional core of the ASD. By contrast, *F. johnsoniae* ribosomes carrying analogous single substitutions have no apparent defects in translation. Moreover, ribosomes with four or five substitutions within the 1,535–1,539 region retain substantial, albeit reduced, activity. These data illuminate the divergent functional roles for the ASD in Gammaproteobacteria versus Bacteroidia.

## Materials and Methods

### *E. coli* Plasmids and Strains

Plasmid p287MS2 carries the *rrnB* operon downstream from the lambda P_L_ promoter (Youngman et al., 2004). Single mutations were made in the ASD region of p287MS2, generating the plasmids of Table 1 (pMDxx; where “xx” represents a unique number). To test for dominant lethal/negative phenotypes, each pMDxx plasmid was transformed into DH10 (pcI857), and transformants were evaluated for growth at 30°C and 43°C, as described (Samaha et al., 1995; Cochella et al., 2007). To test the ability of the mutant ribosomes to support cell growth, the Δ7 strain SQZ10 was employed (Qin et al., 2007; Quan et al., 2015). Each pMDxx plasmid was transformed into SQZ10, selecting for ampicillin resistance (100 μg/ml). The resulting transformants were grown in liquid media, and cells were spread onto plates containing ampicillin and sucrose (5%), to select against the resident plasmid pHKrrnC-sacB. Successful plasmid replacement was evident by a high frequency of sucrose resistant (and kanamycin sensitive) colonies, and subsequently confirmed by plasmid purification and DNA sequencing (Qin et al., 2007). Unsuccessful plasmid replacement was indicated by a low frequency of sucrose resistant colonies; i.e., more than four orders of magnitude lower than the control (p278MS2) case. Most of these colonies retained kanamycin resistance, and any rare isolates sensitive to kanamycin were found to contain the wild-type 16S rRNA gene, presumably due to homologous recombination.

**TABLE 1:** Systematic mutagenesis of the 3’ end of 16S rRNA in *E. coli*.

Nucleotide	Conservation^a^	Substitution	Plasmid	Dominant negative^b^	Supports growth^c^
A1534	97.9	C	pMD24	−	No
		G	pMD25	−	No
		U	pMD26	−	No
C1535	98.1	A	pMD27	+	No
		G	pMD28	+	No
		U	pMD29	+	No
C1536	98.3	A	pMD14	+	No
		G	pMD15	+++	No
		U	pMD16	+	No
U1537	97.8	A	pMD30	++	No
		C	pMD17	+	No
		G	pMD31	+++	No
C1538	98.4	A	pMD18	++	No
		G	pMD19	+++	No
		U	pMD20	++	No
C1539	98.2	A	pMD21	+++	No
		G	pMD22	++	No
		U	pMD23	+++	No
U1540	98.3	A	pMD42	−	Yes
		C	pMD40	−	No
		G	pMD41	−	Yes
U1541	98.9	A	pMD37	−	Yes
		C	pMD38	−	Yes
		G	pMD39	−	Yes
A1542	16.3	C	pMD43	−	Yes
		G	pMD44	−	Yes
		U	pMD45	−	Yes

a Per cent conservation in Bacteria (Cannone et al., 2002).

b Dominant negative growth phenotypes were assessed in DH10 (pcI857, pMDxx) by spotting 20 µL of cells (10^−4^, 10^−5^, and 10^−6^ dilutions of overnight culture) onto LB, plates and incubating at either 30°C (repressed) or 43°C (derepressed). Results after 24 h of incubation were scored as follows: no effect; +, reduced colony size; ++ evidence for growth only at highest level of inoculation; +++, no growth.

c A test of whether the mutant allele is sufficient to support growth. Yes: The resident plasmid pHKrrnC-sacB of Δ7 strain SQZ10 was successfully replaced by pMDxx. No: The frequency of sucrose resistant colonies in the counterselection step was >4 orders of magnitude lower than the control (p278MS2) case, and these colonies typically retained kanamycin resistance. Any rare isolates sensitive to kanamycin were found to contain the wild-type 16S rRNA, gene, presumably due to homologous recombination.

### *F. johnsoniae* Plasmids and Strains

All *F. johnsoniae* strains (Table 2) were grown on rich CYE medium at 30°C. *F. johnsoniae* plasmids (Table 2) were transformed into *E. coli* strain E726 and then moved into *F. johnsoniae* via tri-parental mating as described previously (McBride and Kempf, 1996).

**TABLE 2:** List of *F. johnsoniae* strains and plasmids.

Name	Description	References
*Strains*		
UW101	wild-type	McBride et al. (2009)
ZAM11	*prfB(-FS)*	This work
ZAM18 (Δ1) *^a^*	*prfB(-FS) ΔrrnF*	This work
ZAM21	UW101 (pZM14)	This work
ZAM23 (Δ2) *^a^*	*prfB(-FS) ΔrrnF ΔrrnA*	This work
ZAM25 (Δ3) *^a^*	*prfB(-FS) ΔrrnF ΔrrnA ΔrrnB*	This work
ZAM26	ZAM25 (pZM14)	This work
ZAM28	ZAM25 (pZM17)	This work
ZAM41	ZAM25 (pZM21; GCUCC)	This work
ZAM42	ZAM25 (pZM22; CAUCC)	This work
ZAM43	ZAM25 (pZM23; CCACC)	This work
ZAM46	ZAM25 (pZM26; GAAGC)	This work
ZAM47	ZAM25 (pZM27; AUUGG)	This work
ZAM49	ZAM25 (pZM31; AAAAA)	This work
ZAM50	ZAM25 (pZM32)	This work
Plasmids		
pSCH710	Shuttle vector with IPTG-inducible promoter	Baez et al. (2019)
pYT313	Suicide vector for allelic replacement in Bacteroidia	Zhu et al. (2017)
pZM06	pSCH710 containing *rrnA*	This work
pZM14	pZM06 with 16S mutation C1451U	This work
pZM17	pSCH710 containing tRNA^Ile^-tRNA^Ala^ genes only	This work
pZM21	pZM14 with ASD sequence GCUCC	This work
pZM22	pZM14 with ASD sequence CAUCC	This work
pZM23	pZM14 with ASD sequence CCACC	This work
pZM26	pZM14 with ASD sequence GAAGC	This work
pZM27	pZM14 with ASD sequence AUUGG	This work
pZM31	pZM14 with ASD sequence AAAAA	This work
pZM32	pZM14 with 16S mutation A1492U	This work

a Colloquial name in parentheses.

Mutations to the *F. johnsoniae* chromosome were made using precise allelic replacement (Zhu et al., 2017). Alleles were cloned into the Bam HI and Sph I restriction sites of the suicide vector pYT313 (Zhu et al., 2017). This vector has two selectable markers, *bla* (expressed in *E. coli*) and *ermF* (expressed in *F. johnsoniae*), as well as the counter-selectable *sacB* gene (expressed in *F. johnsoniae*). Alleles were generated by separately amplifying ∼1 kb regions from the *F. johnsoniae* chromosome both up- and downstream of the target site. These two fragments were then inserted into pYT313 using Gibson Assembly (Gibson et al., 2009). Resulting plasmids were moved into *F. johnsoniae*, and erythromycin (Em, 100 μg/ml) resistant transconjugants were selected. Colonies were then screened for plasmid integration at the appropriate chromosomal locations using PCR. Confirmed recombinants were then grown overnight in the absence of Em, to allow for loss of the plasmid via a second recombination event, and then cells were plated on 5% sucrose for counterselection. Sucrose resistant/Em sensitive colonies were then screened via colony PCR for replacement of the wild-type allele for the mutant allele. *rrnF* was deleted from *F. johnsoniae* by removal of the chromosomal region 5,118,368 to 5,124,329. *rrnA* was deleted by removal of the chromosomal region 24,082 to 30,164. *rrnB* was deleted by removal of the chromosomal region 49,9700 to 50,6103.

The *F. johnsoniae rrnA* operon (chromosome positions: 29,556–23,688) was cloned into the Bam HI and Sph I restriction sites of expression vector pSCH710 (Baez et al., 2019), downstream of the inducible *ompA* promoter, to generate pZM06. The marker mutation C1451U (phenotypically silent) was introduced into the plasmid-encoded 16S gene, using site-directed mutagenesis, to yield pZM14. Other *rrn* alleles were similarly cloned into pSCH710 using Gibson Assembly. Plasmids were moved into *F. johnsoniae* strains via tri-parental mating and selecting for Em (100 μg/ml) resistance.

### Growth Competition Assays

Overnight cultures of *F. johnsoniae* UW101 and ZAM11 were used to seed fresh CYE medium both separately (wild-type and mutant only) or mixed (∼1:1, eight replicates). Inoculated cultures were grown up and back-diluted 200-fold to seed another culture, a process repeated daily for 36 days. Aliquots were taken from saturated cultures for use as template for PCR to quantify the fraction of *prfB* mutants. Because the allele of the *prfB* mutant is effectively shortened by the removal of a single base, amplification of the *prfB* gene around the frameshift site resulted in two different size PCR products for the mixed cultures. PCR was done with Phusion DNA polymerase (NEB), since this enzyme leaves clean blunt-ended PCR products. Primers prZM53 (TAT​TGT​GGA​GCG​CCT​TGG​TGC​GTT) and prZM55 (ATT​TCG​ATT​AGC​TTG​GCA​TCA​ACG​TC) were used to amplify the *prfB* alleles, producing a 64 bp product for the wild-type allele and 63 bp for the mutant allele. Radiolabeled prZM53 was included in the reaction. Briefly, prZM53 was 5′ end-labeled using *γ*-[^32^P]-ATP and T4 polynucleotide kinase (NEB) and purified from free γ-[^32^P]-ATP by Sephadex G-25 (Amersham Biosciences) chromatography. PCR products were resolved by denaturing 8% PAGE. Gel imaging and quantification were performed with a Typhoon FLA 9000 phosphorimager (GE Healthcare) and associated software (ImageQuant 5.2).

### Computational Analysis of *prfB* Frameshifting Usage

A list of 997 organisms from the orders Cytophagales, Bacteroidales, Chitinophagales, Flavobacteriales, and Sphingobacteriales marked as GTDB species representatives and as NCBI type material were downloaded from GTDB (Parks et al., 2021) (Table S2). 740 of these genome assemblies were successfully downloaded and ARFA (Bekaert et al., 2006) was run on these assemblies with default parameters. A *prfB* gene was identified in 726 of these assemblies. Out of the 524 of the 726, in which ARFA detects a frameshift, we manually inspected all five for which the E-value for the detection of the gene fragment upstream of the frameshift (ORF0) was above 0.05, and identified one [*Weeksella virosa*, which had the highest of all E-values (0.32) for ORF0] that was miscalled by ARFA as frameshifted. We visualized the phylogenetic tree of the 726 organisms with a detected *prfB* gene using iTOL (Letunic and Bork, 2021).

### Growth Measurements of *F. johnsoniae* Strains

For each strain, cells from overnight cultures were diluted 100-fold into CYE medium. If included, erythromycin was added to a final concentration of 100 μg/ml and IPTG was added to a final concentration of 1 mM. Cultures were shaken at 250 rpm at 30°C, and aliquots were regularly taken throughout growth to measure the optical density (OD) at 600 nm. Doubling times were determined by fitting the data of the logarithmic phase of growth.

### Sucrose Gradient Sedimentation Analyses

Ribosomal particles were fractionated using methods described previously (Qin and Fredrick, 2013). Briefly, cells were grown to mid-log phase (OD_600_ 0.4–0.7), poured over crushed ice, and harvested via centrifugation. The cell pellet was resuspended in lysis buffer [10 mM Tris-HCl (pH 8.0), 10 mM MgCl_2_, 1 mg/ml lysozyme] and flash frozen three times in liquid nitrogen to lyse the cells. Deoxycholate was added (0.3% final), cell debris was pelleted, and clarified lysate (0.4 ml) was loaded onto an 11 ml 10–40% (wt/vol) sucrose gradient and subjected to ultracentrifugation for 3.5 h at 35,000 rpm in an SW41 rotor (Beckman Coulter). Gradients were pumped using a syringe-pump system (Brandel) with in-line UV absorbance detector (UA-6, ISCO; 254 nm), and 1 ml fractions were collected.

Ribosomes were precipitated from sucrose fractions with ethanol, pelleted, and dissolved in 200 µL extraction buffer [0.3 M sodium acetate (pH 6.5), 0.5% SDS, 5 mM EDTA]. RNA was extracted twice with water-saturated phenol and twice with CHCl_3_/isoamyl alcohol (24:1). Extracted RNA was then precipitated with ethanol, pelleted, and dissolved in water.

To determine the relative amount of mutant 16S rRNA in each fraction, poison primer extension was used as described (Abdi and Fredrick, 2005). Primer prZM66 (GTT​ACC​AGT​TTT​ACC​CTA​GGC​A) was designed to anneal to 16S rRNA at a position 3′ of the marker mutation C1451U such that extension of the primer in the presence of dideoxyadenosine triphosphate (ddATP) results in distinct extension products that reflect the fraction of templates containing the mutation. Briefly, prZM66 was 5′ end-labeled using *γ*-[^32^P]-ATP and T4 polynucleotide kinase (NEB) and purified from free *γ*-[^32^P]-ATP by Sephadex G-25 (Amersham Biosciences) chromatography. In a 10-μL reaction containing 50 mM HEPES (pH 7.6) and 100 mM KCl, labeled primer was annealed to ∼1.5 pmol 16S rRNA by heating the reaction to 95°C for 1 min and then allowing it to cool slowly. After a brief centrifugation to recover condensation, 10 μL of 2X extension mix [260 mM Tris-HCl (pH 8.5), 20 mM MgCl_2_, 20 mM DTT, 6 U AMV reverse transcriptase (Life Sciences Advance Technologies Inc.), 340 μM of ddATP, and 340 μM of each other deoxynucleotide triphosphate) was added and the reaction was incubated for 10 min at 42°C. Finally, the primer extension products were passed through a Sephadex G-25 column, dissolved in loading solution (95% formamide, 20 mM EDTA, 0.05% xylene cyanol FF, and 0.05% bromophenol blue), and resolved by denaturing 8% PAGE. Gels were then dried and imaged as described above.

## Results

### Systematic Mutagenesis of the 3’ End of *E. coli* 16S rRNA

The ASD region of the *E. coli* ribosome has been targeted in several previous studies (Hui and de Boer, 1987; Jacob et al., 1987; Lee et al., 1996; Rackham and Chin, 2005; Hui et al., 1988; Yassin et al., 2005). Most of these studies aimed to generate functionally orthogonal ribosomes and hence entailed the simultaneous substitution of multiple nucleotides (e.g., 1,535–1,540). While certain single mutations have been analyzed (Jacob et al., 1987; Yassin et al., 2005), to our knowledge no one has performed a comprehensive analysis of single substitutions across this critical region. We did so here, targeting nine positions (1,534–1,542) of the 16S rRNA gene in plasmid p287MS2 (Youngman et al., 2004). This plasmid contains the ribosomal RNA operon *rrnB* downstream from the P_L_ promoter, allowing temperature-dependent transcription in cells containing a labile form of lambda repressor (cI857). Each plasmid was moved into *E. coli* strain DH10 (pcI857) (Samaha et al., 1995; Durfee et al., 2008), and cell growth was assessed at 43°C, conditions of P_L_ de-repression (Table 1, Supplementary Figure S1). Production of 16S rRNA substituted at position 1,535, 1,536, 1,537, 1,538, or 1,539 conferred dominant negative effects. Certain variants (C1536G, U1537G, C1538G, C1539A, and C1539U) were especially deleterious. Presumably, these strong effects stem from altered specificity of the mutant ribosomes during initiation, consistent with widespread proteomic changes seen in analogous studies (Jacob et al., 1987). Next, each plasmid was tested for its ability to support the growth of SQZ10, an *E. coli* strain lacking all seven chromosomal *rrn* operons (Δ7) (Qin et al., 2007; Quan et al., 2015). Most alleles substituted at positions 1,540–1,542 were able to complement the Δ7 strain, whereas alleles with any mutation further upstream could not (Table 1). Collectively, these data indicate the functional importance of nucleotides 1,534–1,539 in *E. coli* and suggest that CCUCC represents the core ASD.

### Deletion of Three *rrn* Operons Slows the Growth of *F. johnsoniae*

**FIGURE 1:**
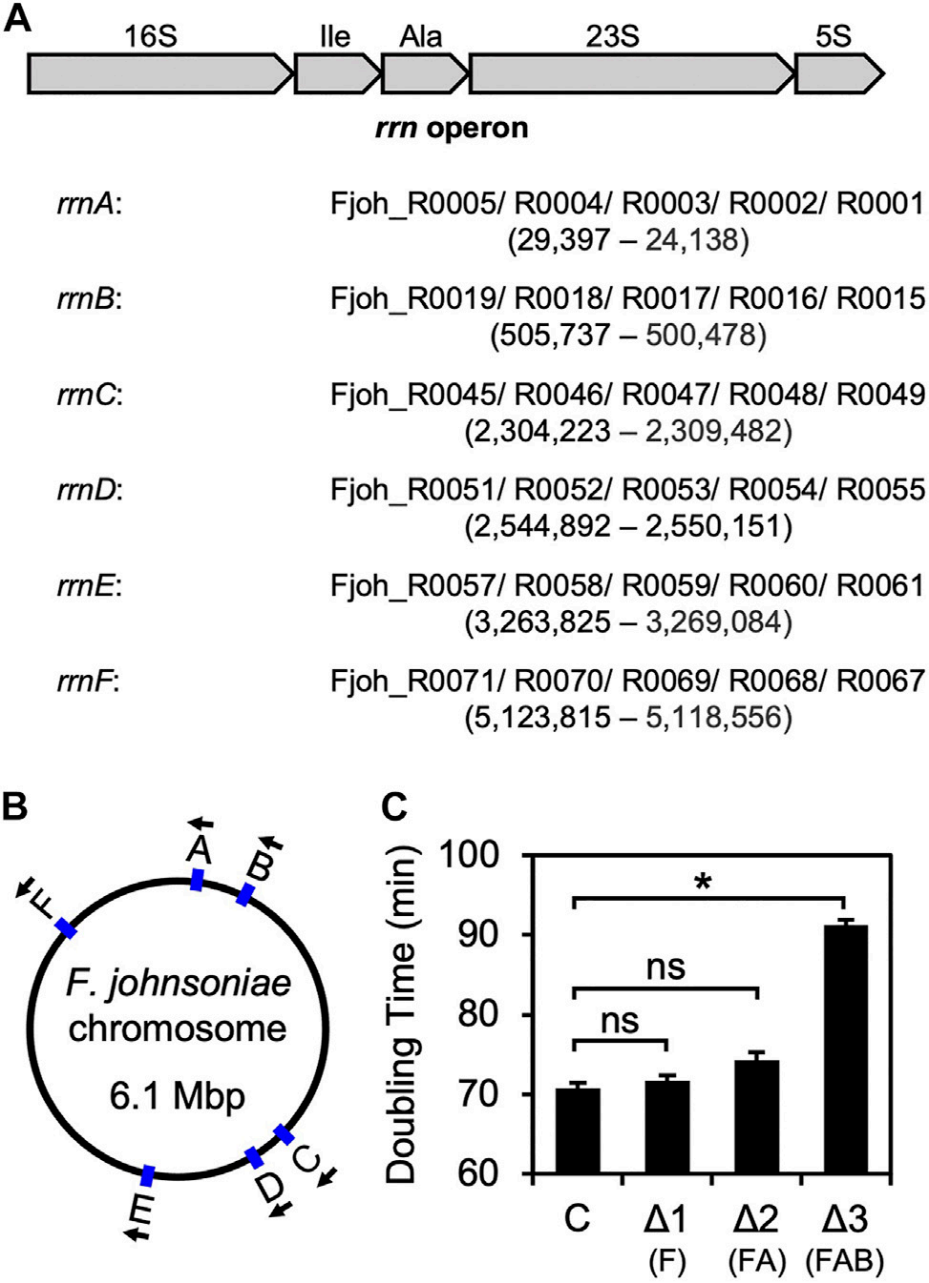
Sequential deletion of *rrn* operons in *F. johnsoniae* **(A)** Common architecture of an *rrn* operon in *F. johnsoniae*. All six operons are virtually identical and encode 16S rRNA (16S), tRNA^Ile^ (Ile), tRNA^Ala^ (Ala), 23S rRNA (23S), and 5S rRNA (5S). Each of the six *rrn* operons in *F. johnsoniae* were assigned a letter, based on chromosomal position. Accession IDs of the component genes for each operon are listed in order, along with the genomic positions (from annotated 5′ end of 16S to 3′ end of 5S) in parenthesis **(B)** A map of the *F. johnsoniae* chromosome with the positions and orientation of each *rrn* operon indicated **(C)** Doubling time (minutes) of the control strain ZAM11 and its deletion derivatives. Data represent the mean ± SEM of three or more independent experiments. Asterisk denotes a significant difference, *p* < 0.05, based on a two-tailed *t* test with the Bonferroni multiple-test correction. ns, not significant. C, control; Δ1, Δ*rrnF*; Δ2, Δ*rrnF*Δ*rrnA*; Δ3, Δ*rrnF*Δ*rrnA*Δ*rrnB.*

On its single chromosome, *F. johnsoniae* contains six virtually identical *rrn* operons that each encode 16S rRNA, tRNA^Ile^ (anticodon GAU), tRNA^Ala^ (anticodon UGC), 23S rRNA, and 5S rRNA. Starting with ZAM11, a strain which constitutively produces RF2 (see below), we began to progressively delete *rrn* operons (named *rrnA-F*, based on chromosome position; Figure 1). Loss of one (*ΔrrnF*) or two (*ΔrrnFΔrrnA*) operons had little if any effect on growth (Figure 1C, Supplementary Table S1). However, loss of three operons (*ΔrrnFΔrrnAΔrrnB*) slowed growth considerably, increasing the doubling time from 70 to 90 min. These findings are reminiscent of *E. coli* studies which showed that a minimum of four chromosomal operons are needed to sustain rapid growth (Quan et al., 2015).

**FIGURE 2:**
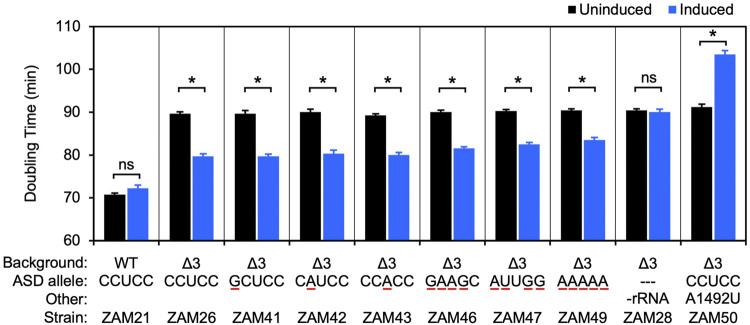
Ribosomes carrying multiple ASD substitutions retain substantial activity *in vivo*. Plasmid pZM14 or one of its derivatives was moved into UW101 (WT) or ZAM25 (Δ3), and cell doubling time in the absence (black bars) or presence (blue bars) of IPTG was measured. The ASD sequence of the plasmid-encoded 16S rRNA is indicated (ASD allele), and substituted nucleotides are underscored in red. In the case of strain ZAM28, only tRNA^Ile^ and tRNA^Ala^ are expressed from the plasmid (-rRNA). Data represent the mean ± SEM of three or more independent experiments. Asterisks denote significant differences, *p* < 0.05, based on a two-tailed *t* test with the Bonferroni multiple-test correction. ns, not significant.

**FIGURE 3:**
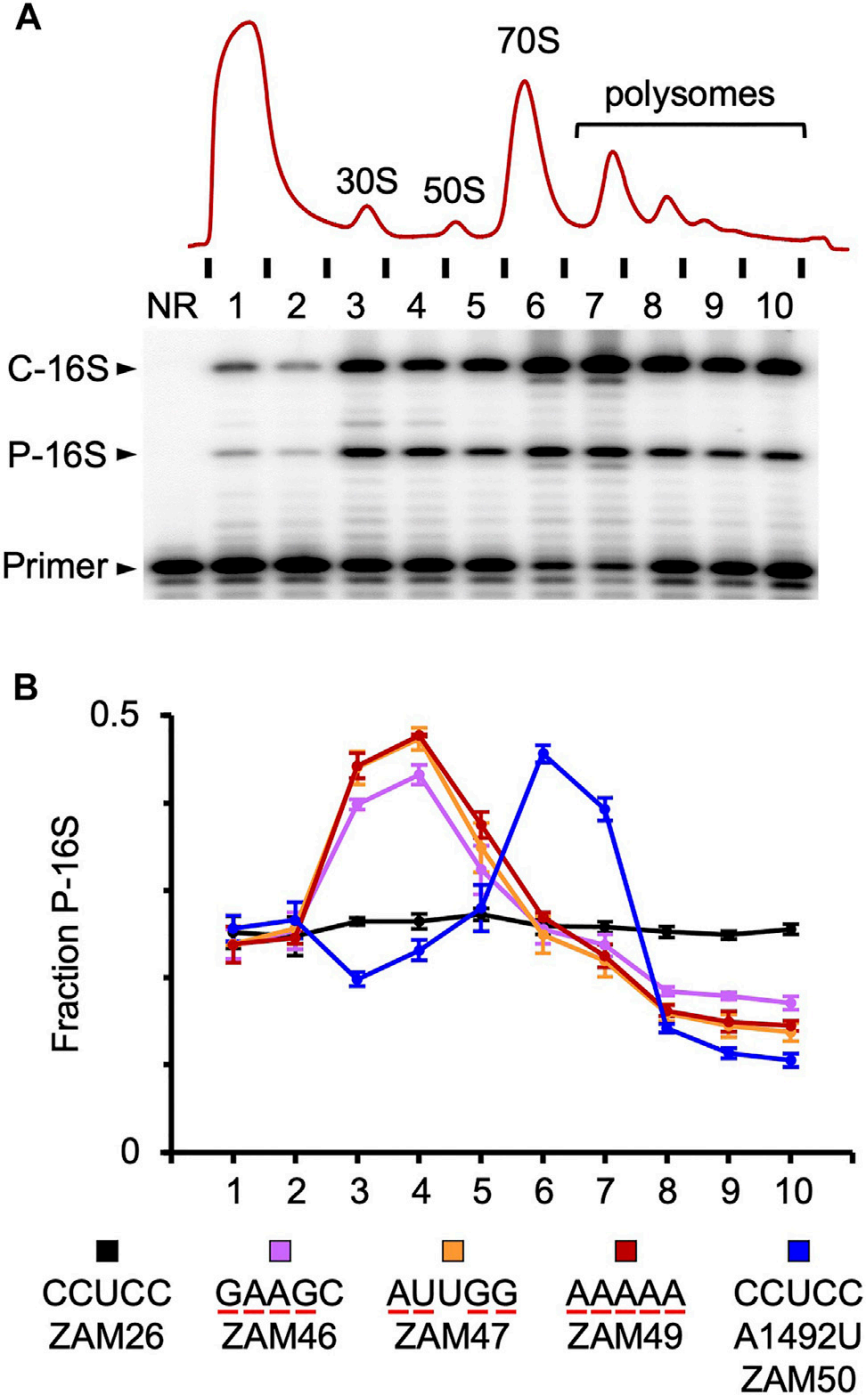
Tracking mutant particles in various ribosomal fractions **(A)** A representative experiment in which ribosomal complexes from induced ZAM26 cells were separated by sucrose gradient sedimentation. RNA isolated from collected fractions (one to ten) was subjected to primer extension analysis to quantify chromosomally-encoded (C-16S) and plasmid-encoded (P-16S) 16S rRNA. NR, no RNA **(B)** The fraction of plasmid-encoded 16S rRNA (P-16S) is plotted as a function of sucrose gradient fraction for various strains (as indicated). For each strain, the experiment was repeated three times, and data points and error bars represent mean ± SEM values. The ASD sequence of the plasmid-encoded 16S rRNA is indicated in the key, and substituted nucleotides are underscored in red. Parallel primer extension analysis of RNA from total (unfractionated) lysates yielded the following values: ZAM26, 0.28 ± 0.01; ZAM46, 0.27 ± 0.01; ZAM47, 0.26 ± 0.01; ZAM49, 0.27 ± 0.01; ZAM50, 0.31 ± 0.01.

An *rrn* operon with a marker mutation (C1451U; a base substitution in the tetraloop of h44, predicted to be phenotypically silent) was cloned downstream of an engineered IPTG-inducible promoter in the shuttle vector pSCH710. The resulting plasmid (pZM14) was moved into the Δ3 strain of *F. johnsoniae*, and growth in the absence and presence of IPTG was measured (Figure 2, ZAM26; Supplementary Table S1). The doubling time decreased from 90 to 80 min in the presence of inducer (1 mM), indicating clear albeit partial complementation by the plasmid-borne *rrn* operon. Expression of tRNA^Ile^ and tRNA^Ala^ only (Figure 2, ZAM28) had no effect, indicating the importance of rRNA in the complementation. The marker mutation C1451U allowed us to track the plasmid-encoded 30S subunits in various ribosome fractions, using primer extension with dideoxy-ATP (Figure 3, ZAM26). These subunits accounted for ∼25% of the total and were distributed evenly across all fractions of the sucrose gradient. This distribution pattern shows that the plasmid-encoded subunits are as active as chromosomally-encoded subunits in these cells.

### 30S Subunits Carrying Single Mutations in the Core ASD Appear to Be Fully Functional in *F. johnsonia*e

Using this complementation system, we began to evaluate mutations to the ASD core. Derivatives of pZM14 harboring various single mutations were made and introduced into the Δ3 strain. Growth of the resulting strains (ZAM41-43) in the absence and presence of IPTG was measured, and the data were indistinguishable from that of the ZAM26 control (Figure 2, Supplementary Table S1). In other words, the mutant 30S subunits carrying C1535G, C1536A, or U1537A rescued the growth defect of the Δ3 strain as well as the control subunits and hence have similar activity. Notably, these same mutations confer dominant negative phenotypes in *E. coli* (Table 1).

As a separate control, we introduced the A-site mutation A1492U into plasmid pZM14 and moved the resulting plasmid into the Δ3 strain (Figure 2, ZAM50; Table S1). Mutation A1492U targets the 30S A site and eliminates translation activity in *E. coli* (Abdi and Fredrick, 2005). Expression of 16S (A1492U) rRNA in the presence of IPTG strongly inhibited growth, increasing the doubling time to 104 min. This dominant negative phenotype is in line with analogous experiments done in *E. coli* (Powers and Noller, 1990; Cochella et al., 2007) and indicates that, in this *F. johnsoniae* system, plasmid-encoded 16S rRNA is expressed at levels high enough to confer such phenotypes.

### 30S Subunits Carrying Multiple Mutations in the Core ASD Retain Substantial Activity in *F. johnsoniae*

Next, we heavily mutagenized the ASD and tested the ability of the corresponding mutant ribosomes to restore growth of the Δ3 strain (Figure 2, Supplementary Table S1). Production of subunits with quadruple substitutions within the ASD (CCUCC to GAAGC or AUUGG; mutations underscored) reduced doubling times from 90 to ∼82 min, rescues nearly as robust as that provided by control (CCUCC) subunits. Subunits in which the core ASD is replaced with AAAAA also stimulated growth, albeit to smaller degree. Thus, ribosomes lacking the ASD sequence can translate endogenous mRNA in *F. johnsoniae*.

### Mutant 16S rRNA Is Enriched in 30S Particles, Indicating Some Defect in Assembly or Initiation

The activity of various mutant ribosomes in the cell was evaluated by quantifying the proportion of plasmid-encoded 16S rRNA in ribosomal particles fractionated by sucrose gradient sedimentation (Figure 3). Subunits carrying four or five substitutions in the ASD exhibited similar profiles, with overrepresentation in the subunit region (fractions 3–4) and underrepresentation in the polysome region (fractions 8–10). These data indicate that wild-type ribosomes outcompete the mutant ribosomes for mRNA loading. This could stem from a defect in initiation or assembly, as in either case 30S particles would accumulate and fewer ribosomes would enter the actively-translating pool. Notably, A_260_ traces of the lysates were similar across the board (Supplementary Figure S2), indicating little or no effects on the overall proportions of subunits, monosomes, and polysomes in these strains.

In the case of A1492U, mutant particles accumulated in the 70S region (fractions 6–7) and were present at reduced levels in both the subunit and polysome regions (Figure 3). As this mutation targets an A-site residue critical for decoding, these mutant ribosomes are presumably trapped as 70S initiation complexes, unable to transition to elongation. Their presence in polysomes can be explained by one or more wild-type ribosomes downstream on the mRNA, and their relative underrepresentation in the subunit region might be explained by an inability of “stuck” 70S complexes to dissociate.

### Elimination of RF2 Autoregulation in *F. johnsoniae* has No Obvious Effect on Cell Fitness

In many bacteria, the gene encoding RF2, *prfB*, is autoregulated via a +1 programmed frameshifting mechanism (Craigen and Caskey, 1986; Weiss et al., 1987; Baranov et al., 2002). In *E. coli*, the frameshift site corresponds to **AGGGGGU**AUCUU ***UGA*** C, where the slippery sequence (underscored) overlaps the in-frame stop codon (bold italics), just downstream from a SD-like sequence (bold). When RF2 levels are low in the cell, ribosomes containing peptidyl-tRNA^Leu^:CUU in the P site and codon UGA in the A site pause. Because the SD-like sequence is closely juxtaposed to the P codon, pairing to the ASD causes tension on the mRNA that promotes slippage of peptidyl-tRNA^Leu^ from CUU (zero frame) to UUU (+1 frame) (Devaraj and Fredrick, 2010). Continued translation in the +1 frame allows production of full-length RF2. As RF2 levels rise, the rate of termination at the in-frame UGA increases, down-regulating further production of the factor.

One might expect Bacteroidia to lack this autoregulatory mechanism because it depends on mRNA-rRNA pairing. However, we noticed that the *prfB* gene of *F. johnsoniae* contains a +1 programmed frameshift site, virtually identical to that of *E. coli* but with a “perfect” SD-like sequence: **AGGAGGU**AUCUU ***UGA*** C (annotated as above). To evaluate the prevalence of *prfB* programmed frameshifting across the class, we used the tool ARFA (Bekaert et al., 2006). Over 700 representative genomes were analyzed, and in 72% of the cases (523/726), *prfB* contains the frameshift. This value is in line with frequencies calculated previously (70–87%), using organisms from multiple phyla (Baranov et al., 2002; Bekaert et al., 2006). Thus, the autoregulatory mechanism seems no less common in the Bacteroidia. Supplementary Figure S3 shows occurrences of programmed frameshifting projected onto the GTDB phylogenetic tree (Parks et al., 2021). The frameshift is present in all Sphingobacteriales analyzed and absent from all Weeksellaceae analyzed. However, the other clades show considerable variability, implying evolutionary loss and/or gain of the autoregulatory mechanism.

**FIGURE 4:**
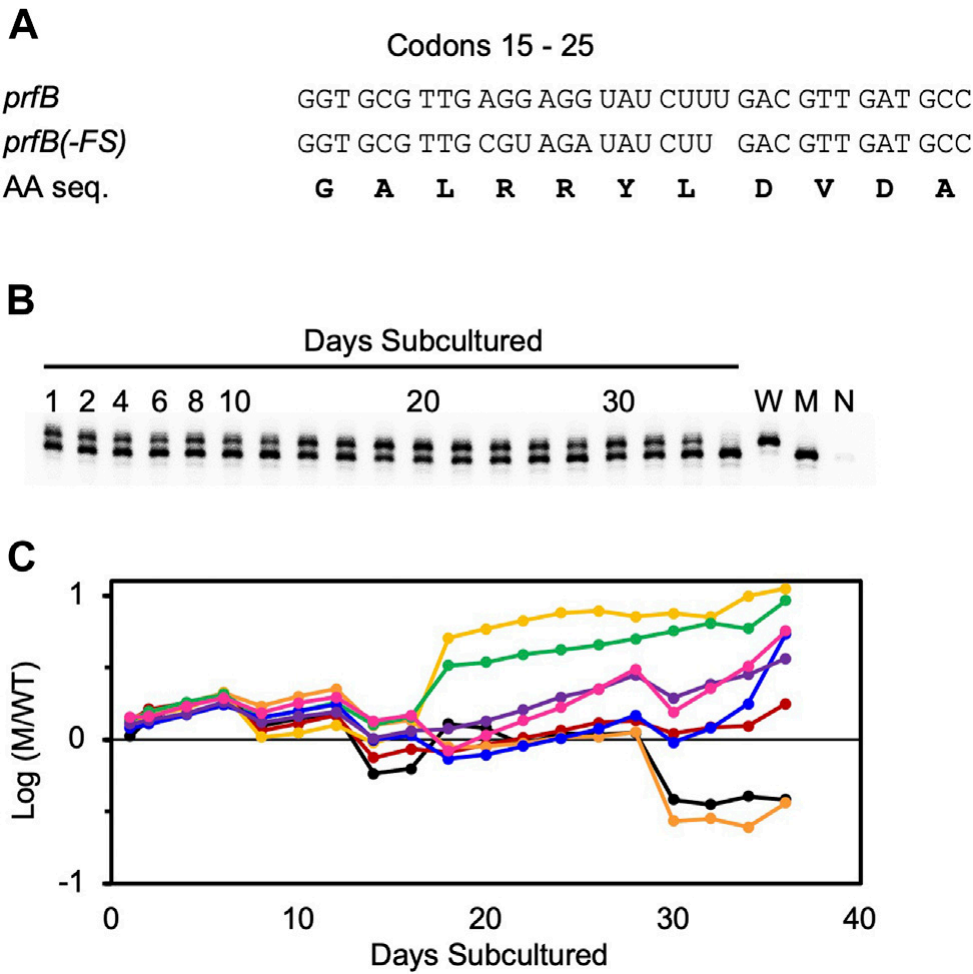
Autoregulation of RF2 synthesis provides no obvious fitness benefit in *F. johnsoniae*. Growth competition between strains UW101 and ZAM11 **(A)** Sequence of codons 15–25 of *prfB* in the wild-type strain UW101 (*prfB*) and the engineered strain ZAM11 (*prfB(-FS)*). Note that the encoded protein sequence (AA seq.) remains unchanged **(B)** An example of the primary data. Mixed cultures were passaged daily, and aliquots were taken and analyzed by PCR to distinguish wild-type and mutant alleles. Control reactions containing pure wild-type cells (W), pure mutant cells (M), or no cells (N) are shown in the rightmost lanes **(C)** The proportion of mutant (M) versus wildtype (W) cells is plotted as a function of time, for eight biological replicates. Each replicate is represented by a different color, and Y values correspond to log_10_ (M/W).

Before embarking on genetic analysis of the ASD (described above), we decided to remove the *prfB* frameshift, because we were mainly interested in roles of the ASD beyond this autoregulatory mechanism. We replaced the frameshift site (codons 18–22) with the sequence CGT AGA TAT CTT GAC. The resulting strain, ZAM11, which contains an undisrupted *prfB* open reading frame encoding the wild-type RF2 protein, exhibited no obvious growth defect. To further characterize the strain, we co-cultured ZAM11 and its parent strain UW101 in eight replicate experiments, growing the cells in CYE medium and passaging them every day for 36 days (Figure 4). Samples were removed at each passage, and PCR was used to quantify the abundance of ZAM11 versus UW101. The ZAM11/UW101 ratio increased or decreased slowly as a function of time, depending on the particular replicate and time window. In six of the eight experiments, ZAM11 predominated by day 36. Hence, this mutation, which effectively removes *prfB* autoregulation, confers no obvious loss of fitness, at least under these laboratory conditions.

## Discussion

In this study, we show that the ASD plays a much smaller role in *F. johnsoniae* than in *E. coli*. In *F. johnsoniae*, ribosomes carrying single mutations at positions 1,535–1,537 are as active as WT ribosomes, based on genetic complementation. In *E. coli*, the same mutations cause a dominant negative or dominant lethal phenotype. Remarkably, *F. johnsoniae* ribosomes retain substantial activity even after quadruple mutation or complete replacement of the ASD (nucleotides 1,535–1,539). This clear difference in ASD dependence can be explained by SD usage in the two organisms. Most *E. coli* genes contain a SD (Nakagawa et al., 2010; Nakagawa et al., 2017), whereas very few *F. johnsoniae* genes do (Jha et al., 2021). In fact, sequences complementary to the 3’ end of 16S rRNA are underrepresented upstream of start codons in *F. johnsoniae* (and other Bacteroidia), implying that rRNA-mRNA pairing can be inhibitory for initiation on many mRNAs (Jha et al., 2021).

*F. johnsoniae* ribosomes carrying multiple ASD mutations are active but functionally compromised. This reduced activity could stem from a defect in 30S assembly. Era, a conserved bacterial GTPase critical for 30S biogenesis, interacts directly with nucleotides 1,530–1,539 of 16S rRNA, which includes the core ASD (nt 1,535–1,539) (Sharma et al., 2005; Tu et al., 2009; Tu et al., 2011; Razi et al., 2019). The mutations we made are predicted to disrupt Era-rRNA contacts, which may slow assembly of the mutant subunits, leading to their enrichment in the 30S region of the gradient and their depletion from the polysome region. Another possibility is that these mutant ribosomes are defective in translation initiation. A recent cryo-EM structure of the *F. johnsoniae* ribosome revealed that the 3′ tail of 16S rRNA binds a pocket formed by bS21, bS18, and bS6 on the 30S platform, explaining why *F. johnsoniae* ribosomes are “blind” to SD sequences *in vivo* and *in vitro* (Jha et al., 2021). Mutations at positions 1,537–1,539 are predicted to disrupt ASD interactions with bS21 and/or bS18 on the platform, effectively liberating the 3’ tail. This might be generally detrimental to *F. johnsoniae* initiation, which normally does not entail mRNA-rRNA pairing. Further work will be needed to distinguish whether these ASD mutations influence assembly or initiation (or both), and exactly how they do so.

Translation of *rpsU* in *F. johnsoniae* does involve SD-ASD pairing (Jha et al., 2021), hence bS21 production may be compromised in cells with ASD-substituted ribosomes. While this could impair 30S assembly, both mutant and wild-type subunits would be equivalently affected, which is inconsistent with our gradient sedimentation results. Instead, our data provide strong evidence that the mutant subunits are specifically defective (Figure 3), while global translation in the cell is largely unchanged (Supplementary Figure S2).

Growth of *F. johnsoniae* slowed substantially after deletion of the third (of six) ribosomal RNA (*rrn*) operons. This is reminiscent of analogous experiments in *E. coli*, where a clear drop in growth occurred upon deletion of the fourth (of seven) operons (Quan et al., 2015). However, the basis of this growth inhibition differs in the two systems. For *E. coli* Δ4, the growth phenotype can be largely rescued by plasmid ptRNA67, which encodes the various tRNA genes associated with seven *rrn* operons, and the additional presence of a plasmid expressing ribosomal RNA has no further effect. In fact, deletion of two more *rrn* operons (strain Δ6) are needed before growth becomes limited by rRNA levels. For the Δ3 strain of *F. johnsoniae*, complementation depends on plasmid-encoded rRNA, not tRNA (Figure 2, ZAM28). In other words, the three intact *rrn* operons on the chromosome are unable to maintain sufficient levels of rRNA in the cell. This hints that feedback regulation of rRNA synthesis in *F. johnsoniae* differs from that in *E. coli* (Paul et al., 2004), a hypothesis worth exploring in the future.

While pZM14 can complement the Δ3 strain of *F. johnsoniae*, the growth rate of ZAM26 does not reach that of wild-type cells. Why only partial complementation is seen remains unclear. One possibility is that production of rRNA from pZM14 is simply not high enough. This plasmid contains the *rrn* operon (*rrnA*) downstream from an engineered *ompA* promoter (Baez et al., 2019), which is probably less active than the native *rrn* promoter. In earlier work (Boleratz, 2016), we cloned the *rrn* operon with its native promoter into an analogous shuttle vector, but we were unable to move the resulting plasmid into *F. johnsoniae*. Further investigation, involving conjugation of numerous deletion derivatives, showed that the *rrn* promoter itself prevents transconjugant formation (Boleratz, 2016). We suspect that high-level transcription directed by P_*rrn*_ interferes with plasmid replication or stability. Unfortunately, these experiments left us with no useful constructs, and the basis of partial complementation remains unsolved.

All bacteria have two primary release factors—RF1, which recognizes UAA and UAG; and RF2, which recognizes UAA and UGA. In many species, production of RF2 is autoregulated via a +1 programmed frameshifting mechanism, which depends on mRNA-rRNA pairing. In this work, we show that *prfB* autoregulation is also common among Bacteroidia, even though ribosomes of these organisms exhibit an occluded ASD and generally fail to recognize SD sequences (Jha et al., 2021). Presumably, the frameshifting mechanism has adapted in these organisms to account for the altered ASD dynamics. Interestingly, ribo-seq read coverage suggests that ribosomes pause at the slippery site much longer in *F. johnsoniae* than in *E. coli* (Baez et al., 2019). We hypothesize that other *cis*-acting elements and/or trans-acting factors promote this pause, allowing enough time for the ASD to dissociate from the 30S platform and pair with the mRNA. Notably, the *prfB* frameshift appears to be uniformly absent in Weeksellaceae. This clade includes Chryseobacteria and related organisms, whose ribosomes have an alternative ASD (5′-UCUCA-3′) (Jha et al., 2021). This observation is consistent with a critical role for rRNA-mRNA pairing in *prfB* frameshifting and hints that multiple G-C pairs may be needed.

Prior to mutational analysis of the ASD, we removed the frameshift site of *prfB* in *F. johnsoniae*. The resulting strain, ZAM11, constitutively produces the wild-type RF2 protein from one open reading frame. Interestingly, ZAM11 exhibited no obvious phenotype, and growth competition experiments revealed no loss of fitness, at least under the laboratory conditions tested. To our knowledge, analogous work has yet to be performed in *E. coli*, or any other bacterium. The prevalence of the autoregulatory mechanism across Bacteria implies that it must provide some benefit. There is some evidence that overproduction of RF2 can be deleterious, perhaps due to misreading of the tryptophan codon UGG (Abdalaal et al., 2020). Further studies of ZAM11 (and/or analogous strains in other bacteria) will be needed to understand the physiological role of *prfB* autoregulation.

## Data Availability

The original contributions presented in the study are included in the article/Supplementary Material, further inquiries can be directed to the corresponding author.

## References

[B1] Abdalaal H. Pundir S. Ge X. Sanyal S. Näsvall J. (2020). Collateral Toxicity Limits the Evolution of Bacterial Release Factor 2 toward Total Omnipotence. Mol. Biol. Evol. 37 (10), 2918–2930. 10.1093/molbev/msaa129 32437534 PMC7530605

[B2] Abdi N. M. Fredrick K. (2005). Contribution of 16S rRNA Nucleotides Forming the 30S Subunit A and P Sites to Translation in *Escherichia coli* . RNA 11 (11), 1624–1632. 10.1261/rna.2118105 16177132 PMC1370848

[B3] Accetto T. Avguštin G. (2011). Inability of Prevotella Bryantii to Form a Functional Shine-Dalgarno Interaction Reflects Unique Evolution of Ribosome Binding Sites in Bacteroidetes. PLoS One 6 (8), e22914. 10.1371/journal.pone.0022914 21857964 PMC3155529

[B4] Baez W. D. Roy B. McNutt Z. A. Shatoff E. A. Chen S. Bundschuh R. (2019). Global Analysis of Protein Synthesis in Flavobacterium Johnsoniae Reveals the Use of Kozak-like Sequences in Diverse Bacteria. Nucleic Acids Res. 47 (20), 10477–10488. 10.1093/nar/gkz855 31602466 PMC6847099

[B5] Baranov P. V. Gesteland R. F. Atkins J. F. (2002). Release Factor 2 Frameshifting Sites in Different Bacteria. EMBO Rep. 3 (4), 373–377. 10.1093/embo-reports/kvf065 11897659 PMC1084053

[B6] Bekaert M. Atkins J. F. Baranov P. V. (2006). ARFA: a Program for Annotating Bacterial Release Factor Genes, Including Prediction of Programmed Ribosomal Frameshifting. Bioinformatics 22 (20), 2463–2465. 10.1093/bioinformatics/btl430 16895933

[B7] Boleratz B. (2016). Studies of Translation Initiation in Flavobacterium Johnsoniae. M.S. Thesis (Columbus, Ohio, United States: The Ohio State University).

[B8] Cannone J. J. Subramanian S. Schnare M. N. Collett J. R. D'Souza L. M. Du Y. (2002). The Comparative RNA Web (CRW) Site: an Online Database of Comparative Sequence and Structure Information for Ribosomal, Intron, and Other RNAs. BMC Bioinformatics 3, 2. 10.1186/1471-2105-3-2 11869452 PMC65690

[B9] Cochella L. Brunelle J. L. Green R. (2007). Mutational Analysis Reveals Two Independent Molecular Requirements during Transfer RNA Selection on the Ribosome. Nat. Struct. Mol. Biol. 14 (1), 30–36. 10.1038/nsmb1183 17159993

[B10] Craigen W. J. Caskey C. T. (1986). Expression of Peptide Chain Release Factor 2 Requires High-Efficiency Frameshift. Nature 322 (6076), 273–275. 10.1038/322273a0 3736654

[B11] de Smit M. H. van Duin J. (1994). Translational Initiation on Structured Messengers. J. Mol. Biol. 235 (1), 173–184. 10.1016/s0022-2836(05)80024-5 8289239

[B12] Devaraj A. Fredrick K. (2010). Short Spacing between the Shine-Dalgarno Sequence and P Codon Destabilizes Codon-Anticodon Pairing in the P Site to Promote +1 Programmed Frameshifting. Mol. Microbiol. 78 (6), 1500–1509. 10.1111/j.1365-2958.2010.07421.x 21143320 PMC3071715

[B13] Durfee T. Nelson R. Baldwin S. Plunkett G. 3rd Burland V. Mau B. (2008). The Complete Genome Sequence of *Escherichia coli* DH10B: Insights into the Biology of a Laboratory Workhorse. J. Bacteriol. 190 (7), 2597–2606. 10.1128/JB.01695-07 18245285 PMC2293198

[B14] Espah Borujeni A. Channarasappa A. S. Salis H. M. (2014). Translation Rate Is Controlled by Coupled Trade-Offs between Site Accessibility, Selective RNA Unfolding and Sliding at Upstream Standby Sites. Nucleic Acids Res. 42 (4), 2646–2659. 10.1093/nar/gkt1139 24234441 PMC3936740

[B15] Gibson D. G. Young L. Chuang R.-Y. Venter J. C. Hutchison C. A. 3rd Smith H. O. (2009). Enzymatic Assembly of DNA Molecules up to Several Hundred Kilobases. Nat. Methods 6 (5), 343–345. 10.1038/nmeth.1318 19363495

[B16] Hockenberry A. J. Pah A. R. Jewett M. C. Amaral L. A. N. (2017). Leveraging Genome-wide Datasets to Quantify the Functional Role of the Anti-shine-dalgarno Sequence in Regulating Translation Efficiency. Open Biol. 7 (1), 160239. 10.1098/rsob.160239 28100663 PMC5303271

[B17] Hui A. de Boer H. A. (1987). Specialized Ribosome System: Preferential Translation of a Single mRNA Species by a Subpopulation of Mutated Ribosomes in *Escherichia coli* . Proc. Natl. Acad. Sci. 84 (14), 4762–4766. 10.1073/pnas.84.14.4762 2440028 PMC305185

[B18] Hui A. S. Eaton D. H. de Boer H. A. (1988). Mutagenesis at the mRNA Decoding Site in the 16S Ribosomal RNA Using the Specialized Ribosome System in *Escherichia coli* . EMBO J. 7 (13), 4383–4388. 10.1002/j.1460-2075.1988.tb03337.x 2468489 PMC455173

[B19] Hussain T. Llácer J. L. Wimberly B. T. Kieft J. S. Ramakrishnan V. (2016). Large-Scale Movements of IF3 and tRNA during Bacterial Translation Initiation. Cell 167 (1), 133–144. 10.1016/j.cell.2016.08.074 27662086 PMC5037330

[B20] Jacob W. F. Santer M. Dahlberg A. E. (1987). A Single Base Change in the Shine-Dalgarno Region of 16S rRNA of *Escherichia coli* Affects Translation of many Proteins. Proc. Natl. Acad. Sci. 84 (14), 4757–4761. 10.1073/pnas.84.14.4757 2440027 PMC305184

[B21] Jha V. Roy B. Jahagirdar D. McNutt Z. A. Shatoff E. A. Boleratz B. L. (2021). Structural Basis of Sequestration of the Anti-shine-dalgarno Sequence in the Bacteroidetes Ribosome. Nucleic Acids Res. 49 (1), 547–567. 10.1093/nar/gkaa1195 33330920 PMC7797042

[B22] Lee K. Holland-Staley C. A. Cunningham P. R. (1996). Genetic Analysis of the Shine-Dalgarno Interaction: Selection of Alternative Functional mRNA-rRNA Combinations. RNA 2 (12), 1270–1285. 8972775 PMC1369453

[B23] Letunic I. Bork P. (2021). Interactive Tree of Life (iTOL) V5: an Online Tool for Phylogenetic Tree Display and Annotation. Nucleic Acids Res. 49 (W1), W293–W296. 10.1093/nar/gkab301 33885785 PMC8265157

[B24] Li G.-W. Burkhardt D. Gross C. Weissman J. S. (2014). Quantifying Absolute Protein Synthesis Rates Reveals Principles Underlying Allocation of Cellular Resources. Cell 157 (3), 624–635. 10.1016/j.cell.2014.02.033 24766808 PMC4006352

[B25] McBride M. J. Kempf M. J. (1996). Development of Techniques for the Genetic Manipulation of the Gliding Bacterium Cytophaga Johnsonae. J. Bacteriol. 178 (3), 583–590. 10.1128/jb.178.3.583-590.1996 8550486 PMC177698

[B26] McBride M. J. Xie G. Martens E. C. Lapidus A. Henrissat B. Rhodes R. G. (2009). Novel Features of the Polysaccharide-Digesting Gliding Bacterium Flavobacterium Johnsoniae as Revealed by Genome Sequence Analysis. Appl. Environ. Microbiol. 75 (21), 6864–6875. 10.1128/AEM.01495-09 19717629 PMC2772454

[B27] Mimee M. Tucker A. C. Voigt C. A. Lu T. K. (2015). Programming a Human Commensal Bacterium, Bacteroides Thetaiotaomicron, to Sense and Respond to Stimuli in the Murine Gut Microbiota. Cell Syst. 1 (1), 62–71. 10.1016/j.cels.2015.06.001 26918244 PMC4762051

[B28] Nakagawa S. Niimura Y. Gojobori T. (2017). Comparative Genomic Analysis of Translation Initiation Mechanisms for Genes Lacking the Shine-Dalgarno Sequence in Prokaryotes. Nucleic Acids Res. 45 (7), 3922–3931. 10.1093/nar/gkx124 28334743 PMC5397173

[B29] Nakagawa S. Niimura Y. Miura K.-i. Gojobori T. (2010). Dynamic Evolution of Translation Initiation Mechanisms in Prokaryotes. Proc. Natl. Acad. Sci. 107 (14), 6382–6387. 10.1073/pnas.1002036107 20308567 PMC2851962

[B30] Parks D. H. Chuvochina M. Rinke C. Mussig A. J. Chaumeil P.-A. Hugenholtz P. (2021). GTDB: an Ongoing Census of Bacterial and Archaeal Diversity through a Phylogenetically Consistent, Rank Normalized and Complete Genome-Based Taxonomy. Nucleic Acids Res. 10.1093/nar/gkab776 PMC8728215 34520557

[B31] Paul B. J. Ross W. Gaal T. Gourse R. L. (2004). rRNA Transcription in *Escherichia coli* . Annu. Rev. Genet. 38, 749–770. 10.1146/annurev.genet.38.072902.091347 15568992

[B32] Powers T. Noller H. F. (1990). Dominant Lethal Mutations in a Conserved Loop in 16S rRNA. Proc. Natl. Acad. Sci. 87 (3), 1042–1046. 10.1073/pnas.87.3.1042 2405392 PMC53406

[B33] Qin D. Abdi N. M. Fredrick K. (2007). Characterization of 16S rRNA Mutations that Decrease the Fidelity of Translation Initiation. RNA 13 (12), 2348–2355. 10.1261/rna.715307 17942743 PMC2080605

[B34] Qin D. Fredrick K. (2013). Analysis of Polysomes from Bacteria. Methods Enzymol. 530, 159–172. 10.1016/B978-0-12-420037-1.00008-7 24034320

[B35] Quan S. Skovgaard O. McLaughlin R. E. Buurman E. T. Squires C. L. (2015). Markerless *Escherichia coli* Rrn Deletion Strains for Genetic Determination of Ribosomal Binding Sites. G3 (Bethesda) 5 (12), 2555–2557. 10.1534/g3.115.022301 26438293 PMC4683628

[B36] Rackham O. Chin J. W. (2005). A Network of Orthogonal Ribosome·mRNA Pairs. Nat. Chem. Biol. 1 (3), 159–166. 10.1038/nchembio719 16408021

[B37] Razi A. Davis J. H. Hao Y. Jahagirdar D. Thurlow B. Basu K. (2019). Role of Era in Assembly and Homeostasis of the Ribosomal Small Subunit. Nucleic Acids Res. 47 (15), 8301–8317. 10.1093/nar/gkz571 31265110 PMC6736133

[B38] Samaha R. R. Green R. Noller H. F. (1995). A Base Pair between tRNA and 23S rRNA in the Peptidyl Transferase centre of the Ribosome. Nature 377 (6547), 309–314. 10.1038/377309a0 7566085

[B39] Sharma M. R. Barat C. Wilson D. N. Booth T. M. Kawazoe M. Hori-Takemoto C. (2005). Interaction of Era with the 30S Ribosomal Subunit. Mol. Cell 18 (3), 319–329. 10.1016/j.molcel.2005.03.028 15866174

[B40] Shine J. Dalgarno L. (1974). The 3'-terminal Sequence of *Escherichia coli* 16S Ribosomal RNA: Complementarity to Nonsense Triplets and Ribosome Binding Sites. Proc. Natl. Acad. Sci. 71 (4), 1342–1346. 10.1073/pnas.71.4.1342 4598299 PMC388224

[B41] Steitz J. A. Jakes K. (1975). How Ribosomes Select Initiator Regions in mRNA: Base Pair Formation between the 3' Terminus of 16S rRNA and the mRNA during Initiation of Protein Synthesis in *Escherichia coli* . Proc. Natl. Acad. Sci. 72 (12), 4734–4738. 10.1073/pnas.72.12.4734 1107998 PMC388805

[B42] Studer S. M. Joseph S. (2006). Unfolding of mRNA Secondary Structure by the Bacterial Translation Initiation Complex. Mol. Cell 22 (1), 105–115. 10.1016/j.molcel.2006.02.014 16600874

[B43] Tu C. Zhou X. Tarasov S. G. Tropea J. E. Austin B. P. Waugh D. S. (2011). The Era GTPase Recognizes the GAUCACCUCC Sequence and Binds helix 45 Near the 3' End of 16S rRNA. Proc. Natl. Acad. Sci. 108 (25), 10156–10161. 10.1073/pnas.1017679108 21646538 PMC3121871

[B44] Tu C. Zhou X. Tropea J. E. Austin B. P. Waugh D. S. Court D. L. (2009). Structure of ERA in Complex with the 3' End of 16S rRNA: Implications for Ribosome Biogenesis. Proc. Natl. Acad. Sci. 106 (35), 14843–14848. 10.1073/pnas.0904032106 19706445 PMC2736428

[B45] Vellanoweth R. L. Rabinowitz J. C. (1992). The Influence of Ribosome-Binding-Site Elements on Translational Efficiency in Bacillus Subtilis and *Escherichia coli In Vivo* . Mol. Microbiol. 6 (9), 1105–1114. 10.1111/j.1365-2958.1992.tb01548.x 1375309

[B46] Wegmann U. Horn N. Carding S. R. (2013). Defining the bacteroides Ribosomal Binding Site. Appl. Environ. Microbiol. 79 (6), 1980–1989. 10.1128/AEM.03086-12 23335775 PMC3592243

[B47] Weiss R. B. Dunn D. M. Atkins J. F. Gesteland R. F. (1987). Slippery Runs, Shifty Stops, Backward Steps, and Forward Hops: -2, -1, +1, +2, +5, and +6 Ribosomal Frameshifting. Cold Spring Harbor Symposia Quantitative Biol. 52, 687–693. 10.1101/sqb.1987.052.01.078 3135981

[B48] Yassin A. Fredrick K. Mankin A. S. (2005). Deleterious Mutations in Small Subunit Ribosomal RNA Identify Functional Sites and Potential Targets for Antibiotics. Proc. Natl. Acad. Sci. 102 (46), 16620–16625. 10.1073/pnas.0508444102 16269538 PMC1283848

[B49] Youngman E. M. Brunelle J. L. Kochaniak A. B. Green R. (2004). The Active Site of the Ribosome Is Composed of Two Layers of Conserved Nucleotides with Distinct Roles in Peptide Bond Formation and Peptide Release. Cell 117 (5), 589–599. 10.1016/s0092-8674(04)00411-8 15163407

[B50] Zhu Y. Thomas F. Larocque R. Li N. Duffieux D. Cladière L. (2017). Genetic Analyses Unravel the Crucial Role of a Horizontally Acquired Alginate Lyase for Brown Algal Biomass Degradation by Z Obellia Galactanivorans. Environ. Microbiol. 19 (6), 2164–2181. 10.1111/1462-2920.13699 28205313

