# Supplementary material for: Comparative Analysis of anti-Shine- Dalgarno Function in Flavobacterium johnsoniae and Escherichia coli
Source: Front Mol Biosci. 2021 Dec 13;8:787388. doi: 10.3389/fmolb.2021.787388 (PMC8710568; doi:10.3389/fmolb.2021.787388)
Supplement: Supplementary file 2 [file DataSheet1.PDF]

SUPPLEMENTARY MATERIAL

Comparative analysis of anti-Shine Dalgarno function in *Flavobacterium johnsoniae* and *Escherichia coli*

Zakkary A. McNutt[1,2], Mai D. Gandhi[3], Elan A. Shatoff[2,4], Bappaditya Roy[2,3], Aishwarya Devaraj[1,2], Ralf Bundschuh[2,4,5,6], and Kurt Fredrick[1,2,3]

[1]Ohio State Biochemistry Program, The Ohio State University, Columbus, Ohio 43210, USA
[2]Center for RNA Biology, The Ohio State University, Columbus, Ohio 43210, USA
[3]Department of Microbiology, The Ohio State University, Columbus, Ohio 43210, USA
[4]Department of Physics, The Ohio State University, Columbus, Ohio 43210, USA
[5]Department of Chemistry & Biochemistry, The Ohio State University, Columbus, Ohio 43210, USA
[6]Division of Hematology, Department of Internal Medicine, The Ohio State University, Columbus, Ohio 43210, USA

This file contains:

Figure S1
Figure S2
Figure S3
Table S1

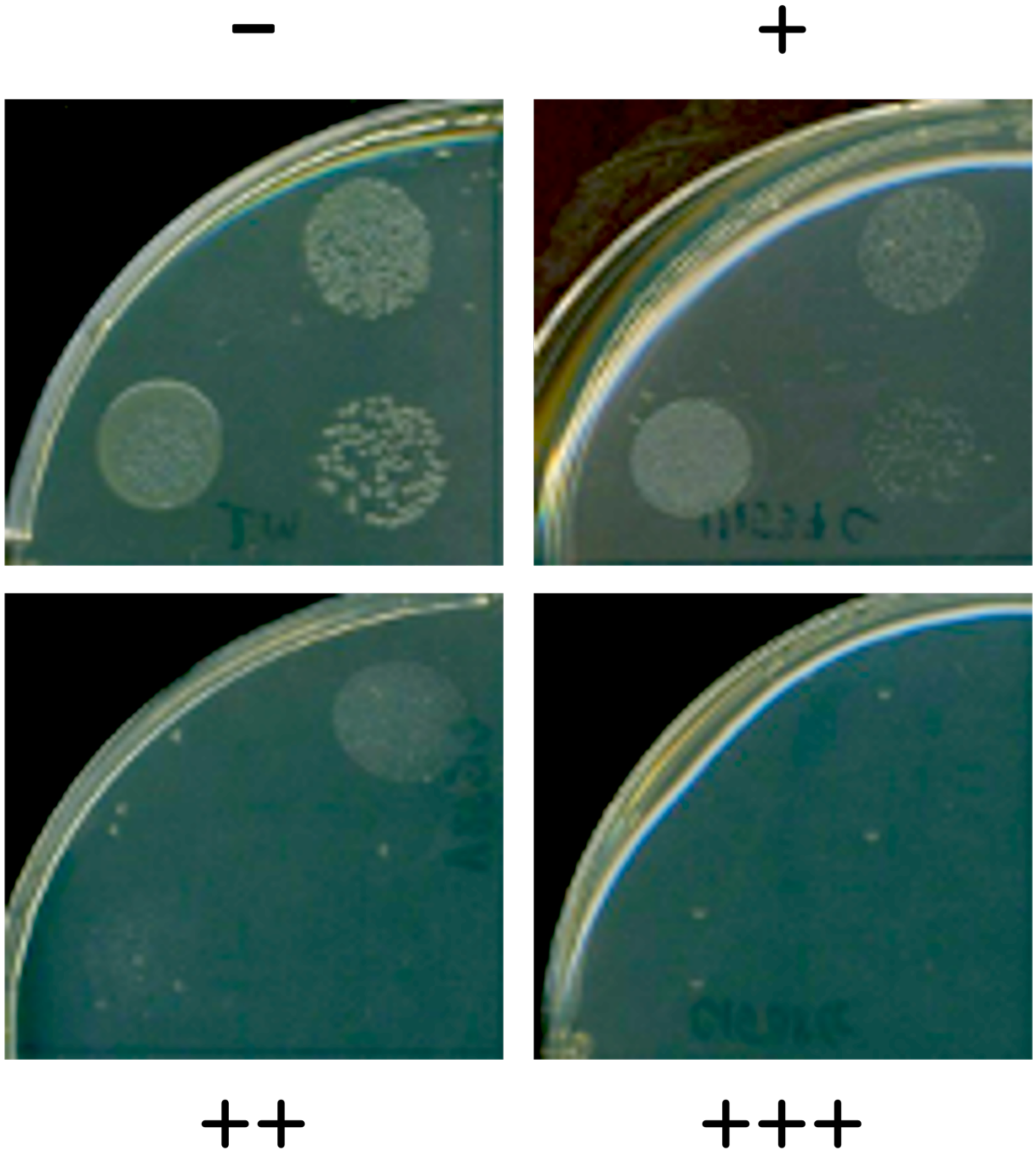

Figure S1. Dominant negative growth phenotypes in *E. coli* were scored based on a plating assay. Shown are plates co-incubated at 43° C that exemplify the degrees of phenotype conferred by expression of mutant 16S rRNA: -, no phenotype; +, reduced colony size; ++ evidence for growth only at highest level of inoculation; +++, no growth.

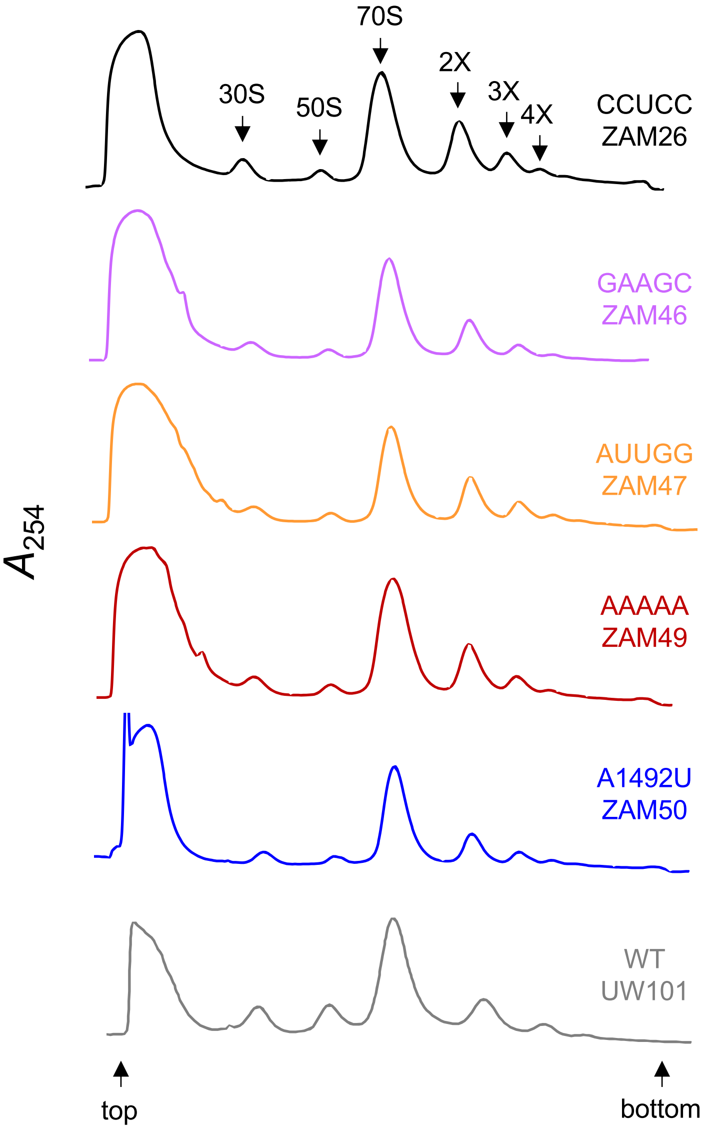

Figure S2.

Sucrose gradient sedimentation analysis of lysates from various *F. johnsoniae* strains. Strains (as indicated) were grown in the presence of IPTG to allow expression of plasmid-borne *rrnA*. Peaks corresponding to subunits (30S, 50S), monosomes (70S), and polysomes (2X, 3X, 4X) are indicated. Arrows on the bottom indicate the top and bottom of the gradient. A_{254}, absorbance at 254 nM.

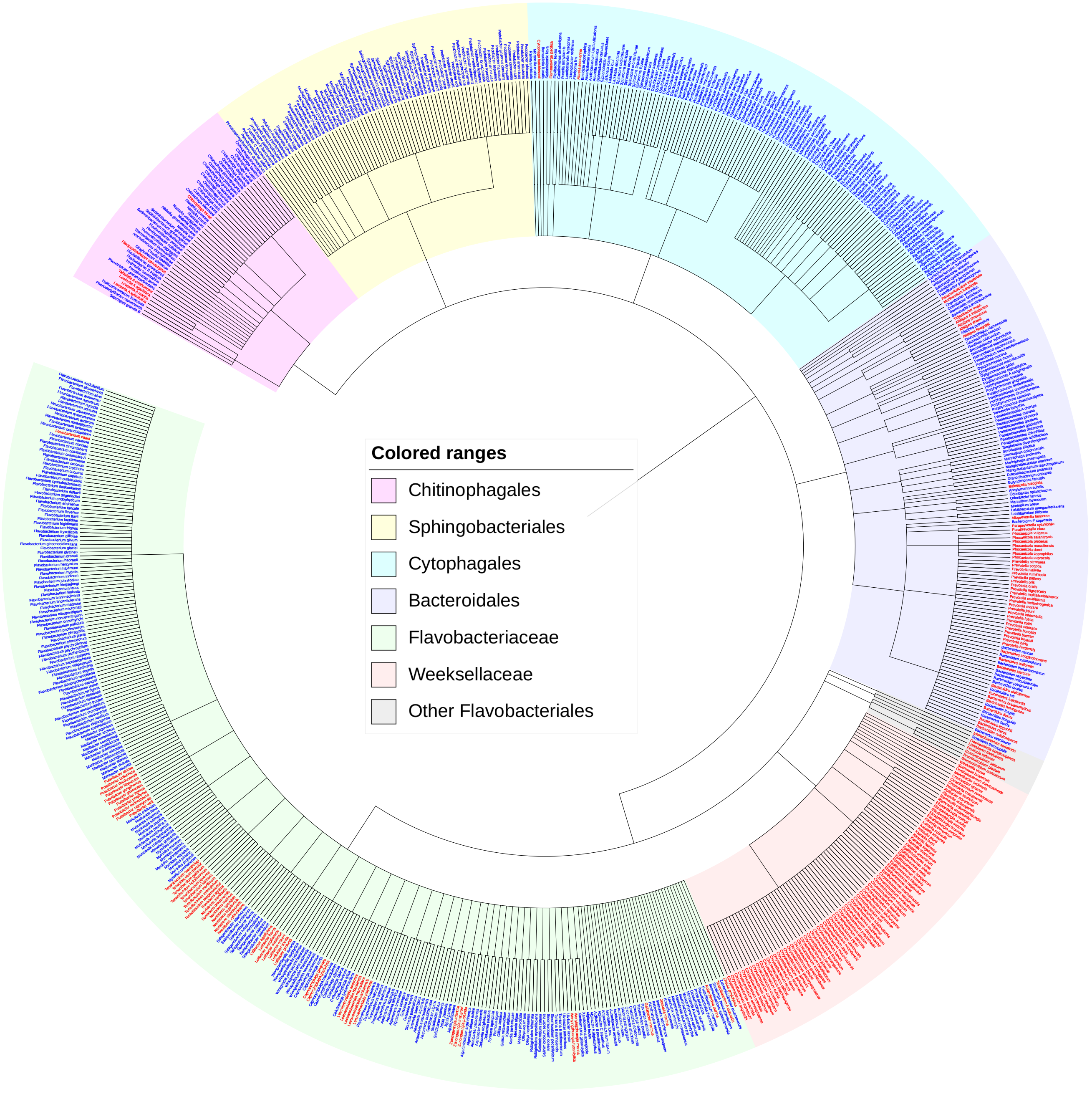

Figure S3.

Scoring the presence of the +1 programmed frameshift site in *prfB* across the Bacteroidia. Shown is a phylogenetic tree of 726 species, which either contain (blue font) or lack (red font) the frameshift. Clades are distinguished by colored shading (as indicated in key).

Table S1. Doubling times measured in this study

Strain	Uninduced	Induced
ZAM11	70.7 ± 0.7	NA
ZAM18	71.7 ± 0.7	NA
ZAM23	74.3 ± 1.0	NA
ZAM25	91.2 ± 0.7	NA
ZAM21	70.7 ± 0.4	72.2 ± 0.8
ZAM26	89.6 ± 0.5	79.7 ± 0.6
ZAM41	89.6 ± 0.8	79.7 ± 0.5
ZAM42	90.0 ± 0.7	80.3 ± 0.8
ZAM43	89.2 ± 0.4	80.0 ± 0.6
ZAM46	90.0 ± 0.5	81.5 ± 0.4
ZAM47	90.3 ± 0.3	82.5 ± 0.4
ZAM49	90.4 ± 0.4	83.5 ± 0.6
ZAM28	90.4 ± 0.4	90.0 ± 0.7
ZAM50	91.2 ± 0.7	103.4 ± 0.9

Data represent the mean ± SEM of 3 or more independent experiments. NA, not applicable.
